# Social Determinants of Pain Prevalence: A Cross-National Comparison of Four European Countries

**DOI:** 10.3390/healthcare14193242

**Published:** 2026-10-01

**Authors:** Teodora Aleksijevic, Vesna Bjegovic-Mikanovic, Stefan Mandic-Rajcevic, Zoran Bukumiric, Biljana Bojic, Ivan Palibrk

**Affiliations:** 1 Faculty of Medicine, University of Belgrade, 11000 Belgrade, Serbia; aleksijevic.teodora@gmail.com (T.A.); stefan.mandic-rajcevic@med.bg.ac.rs (S.M.-R.); zoran.bukumiric@med.bg.ac.rs (Z.B.); bojic.bi1981@gmail.com (B.B.); ivanpalibrk@yahoo.com (I.P.); 2 Clinic for Digestive Surgery, Department of Anesthesiology and Intensive Care, Centre for Anesthesiology and Resuscitation, University Clinical Centre of Serbia, 11000 Belgrade, Serbia

**Keywords:** pain, pain prevalence, social determinants of health, European Health Interview Survey, cross-national study

## Abstract

**Highlights:**

**What are the main findings?**

- Pain prevalence varied markedly across Serbia (35.4%), Bulgaria (41.5%), Norway (43.6%) and Finland (69.9%), confirming that pain is socially patterned and context-dependent.
- In our study, accidents/injuries (OR range, 1.43–2.66), greater daily life limitations (OR range, 1.55–2.52), poorer self-rated health (OR range, 1.66–2.22), and female gender (OR range, 1.38–1.91) were consistently associated with higher odds of pain in all countries, while musculoskeletal disorders showed the largest OR among significant factors in countries where they were retained in the models (OR range, 3.40–4.26). Socioeconomic and behavioural factors showed more context-specific patterns.

**What are the implications of the main findings?**

- Pain prevention and management strategies should extend beyond clinical treatment to address social determinants of health, with approaches tailored to national socioeconomic and health system contexts.
- Cross-national harmonised surveys such as the EHIS provide a valuable and underutilised resource for identifying common and context-specific factors associated with pain at the population level.

**Abstract:**

**Background/Objectives**: Pain is a major public health burden shaped by social and structural factors, yet cross-national evidence on how social determinants influence pain prevalence across socioeconomically diverse European settings remains limited. This study aimed to examine the associations between social determinants and the prevalence of pain in four European countries, and to explore how these associations vary by national context. **Methods**: We conducted a multinational secondary analysis of cross-sectional data from the third wave of the European Health Interview Survey (EHIS), using Eurostat microdata for Serbia, Bulgaria, Norway and Finland (n = 34,882 respondents aged ≥15 years). Pain prevalence was examined in relation to sociodemographic characteristics, health status, chronic conditions and behavioural determinants. LASSO-selected logistic regression models were fitted separately for each country. **Results**: Pain prevalence varied markedly across countries, ranging from 35.4% in Serbia to 69.9% in Finland (*p* < 0.001). Musculoskeletal disorders showed strong associations with pain in countries where this factor was retained in the models (OR range, 3.40–4.26), while accidents/injuries (OR range, 1.43–2.66), greater daily life limitations (OR range, 1.55–2.52), poorer self-rated health (OR range, 1.66–2.22), and female gender (OR range, 1.38–1.91) were consistently associated with higher odds of pain in all countries. The associations of education, employment, income, household composition, and behavioural factors with pain varied across national contexts and may reflect country-specific socioeconomic conditions. **Conclusions**: Pain prevalence was associated with a broad range of social, demographic and health-related factors, with the magnitude and direction of associations varying across national contexts. Pain prevention and management strategies should address both clinical characteristics and the broader social determinants of health, tailored to the national socioeconomic environment.

## 1. Introduction

Chronic pain is one of the most prevalent and burdensome conditions in Europe, yet its distribution across populations is far from random. Growing evidence suggests that the experience of pain is deeply associated with the social conditions in which people live, work and age, making it as much a social phenomenon as a biological one [1,2].

According to the International Association for the Study of Pain (IASP), pain is defined as “an unpleasant sensory and emotional experience associated with, or resembling that associated with, actual or potential tissue damage” [3], acknowledging its inherently subjective character. Depending on the condition examined, the prevalence of pain ranges from approximately 20% to 70% across different patient populations and disease contexts [4]. These figures span a range of definitions and reference periods; the present study uses a four-week reference period consistent with the EHIS instrument, which should be distinguished from chronic pain prevalence estimates based on duration criteria of three months or longer.

Because pain affects biological, psychological and social functioning simultaneously, understanding its determinants requires moving beyond individual pathology toward the broader structural conditions that shape health.

The social determinants of health (the conditions in which people are born, grow, live, work and age, together with economic, political and normative factors) define an individual’s position in society and their access to resources, power and healthcare [5]. Recognising these factors in the context of pain shifts the analytical focus from a purely biomedical model toward a broader framework in which pain is understood as a social experience, associated with systemic and structural forces [6]. Many studies confirm that socioeconomic status, educational attainment and employment are associated with the prevalence, intensity, prognosis and treatment outcomes of pain [7]. Individuals with lower socioeconomic status and educational attainment are disproportionately affected by conditions such as low back pain [8], while social support, social exclusion, trauma and stigmatisation are independently associated with pain-related disability and reduced quality of life [9,10].

Alongside structural and socioeconomic conditions, the biopsychosocial model of pain recognises that psychological processes play a central role in shaping how individuals experience and respond to pain [11]. Social disadvantage does not necessarily influence pain directly; rather, adverse social and health conditions may translate into greater pain burden and interference partly through psychological mechanisms. Among these, pain catastrophizing, a pattern of negative cognitive and emotional appraisal characterised by rumination, magnification and helplessness, has emerged as one of the most consistently identified psychological correlates of pain intensity, disability and emotional consequences. A recent systematic review by Shimada et al. (2026) synthesised evidence from 20 studies and found pain catastrophizing to be robustly associated with pain intensity, disability and pain interference in chronic musculoskeletal pain populations [12]. At the individual level, a longitudinal study in women with endometriosis demonstrated that catastrophizing plays a specific role in amplifying pain interference with daily activities and depressive symptoms over time [13]. These findings suggest that a complete understanding of population-level pain burden requires attention not only to the social conditions that shape pain prevalence but also to the psychological mechanisms through which those conditions translate into functional and emotional consequences.

Large European population studies and meta-analyses have documented pain prevalence rates of 12–48%, with consistent gradients by socioeconomic position and marked variation across regions [14]. However, most comparative research has focused on Western European high-income settings, leaving the mechanisms linking social determinants to pain outcomes across socioeconomically diverse European contexts insufficiently understood. To address this gap, we selected four countries that together represent a broad spectrum of European socioeconomic and health system contexts: Norway and Finland are high-income Nordic welfare states characterised by comprehensive social security networks, universal healthcare coverage and strong equity-oriented health policies [15,16], while Serbia and Bulgaria are middle-income Southeast European countries at different stages of health system transition, with greater socioeconomic inequality and more constrained public resources [17,18,19]. This contrast maximises variability in socioeconomic indicators, gender norms and cultural attitudes toward pain, enabling the identification of both context-specific social determinants of pain across Europe.

The aim of this study was to examine the associations between social determinants and the prevalence of pain in four European countries, and to explore how these associations vary by national context. Specifically, we analysed sociodemographic variables, health status indicators, chronic conditions, and behavioural determinants as factors associated with pain, using data from the third wave of the European Health Interview Survey (EHIS).

## 2. Materials and Methods

### 2.1. Study Design and Data Source

We conducted a multinational secondary analysis of cross-sectional data from the third wave of the European Health Interview Survey (EHIS), accessed via the Eurostat international database. Four countries were selected (Serbia, Bulgaria, Norway and Finland) to represent a broad spectrum of European socioeconomic and health system contexts, spanning northern and southeastern Europe [20]. The EHIS is a harmonised survey designed to assess and compare health status, use of health services, access limitations, and social determinants of health across European countries. The legal basis for EHIS data collection is established by Regulation (EU) No. 1338/2008 and Commission Regulation (EU) No. 2018/255, which mandate the collection of health statistics every five years and define the data specifications for cross-country comparability. Ethical standards in all four countries comply with the Declaration of Helsinki (1964, revised 2013) and applicable national regulations; all respondents provided informed consent to participate in the original survey. Eurostat approval for this secondary analysis was obtained prior to data access (No: RPP 279/2025-EHIS, 1 December 2025) and the Ethical Commission of the Faculty of Medicine, University of Belgrade, approved the secondary analysis (decision No: 20/VII-7).

### 2.2. Population and Sample

The study population comprised residents of Serbia, Bulgaria, Norway and Finland aged 15 years and older. Sampling frames were derived from the most recent national census and population registers. Persons living in institutions were excluded. A total of 34,882 respondents were included in the analysis, comprising 13,178 from Serbia, 7540 from Bulgaria, 7913 from Norway, and 6251 from Finland [20]. Sampling designs differed by country to meet national precision requirements: stratified multiphase sampling was applied in Serbia and Bulgaria (first-stage random selection of census enumeration areas with probability proportional to size, followed by equal-probability household sampling); stratified sampling by territorial area was used in Norway; and simple random sampling with equal probability of inclusion was used in Finland.

Sample attrition occurred in two stages. At Stage 1, respondents with missing data on the primary outcome variable (EHIS item PN1), including both item non-response (code −1, ‘not stated’) and proxy-reported responses (code −3), were excluded from the pain prevalence analysis, since pain is an inherently subjective experience that cannot be reliably assessed by proxy [20,21]. This yielded a pooled prevalence analysis sample of 33,384 respondents (loss of 1498, 4.3% of the enrolled sample). At Stage 2, within each country, respondents with missing data on one or more covariates retained in the LASSO candidate set were excluded from the regression analyses under a complete-case approach, yielding country-specific regression samples of 12,124 (Serbia), 6734 (Bulgaria), 7236 (Norway) and 5193 (Finland). Finland’s higher attrition at this stage (16.9% from the enrolled cohort) may be related to the larger number of variables excluded from Finland’s candidate set due to data unavailability, thereby reducing the number of respondents with complete covariate profiles. The full participant flow and missing data per country are presented in Supplementary Table S1.

### 2.3. Survey Instrument

A standardised questionnaire with a common methodological guide applied across all four countries served as the data collection instrument [21]. A structured translation protocol comprising initial translation by a health statistics expert, expert review, and cognitive testing was used to ensure cross-national comparability of health concepts.

The EHIS third-wave questionnaire comprises three thematic modules: (1) health status, (2) use of health services, and (3) determinants of health, covering 21 sub-modules and a section on key social variables.

The health status module (Module 1) includes self-rated health (assessed on a five-point scale collapsed to very good/good, fair, and bad/very bad), long-standing health problems, daily life limitations, and the presence of chronic conditions (cardiovascular, chronic respiratory, musculoskeletal, endocrine and metabolic, mental health, and genitourinary diseases, as well as accidents and injuries). Pain is assessed as a separate chapter within Module 1: respondents rate pain intensity over the preceding four weeks on a five-point scale (none, very mild/mild, moderate, severe, very severe), and the degree to which pain interferes with daily activities on a five-point scale (not at all, a little bit, moderately, quite a bit, extremely).

The second module (Module 2) addresses healthcare utilization and access to health services; as this manuscript focuses exclusively on pain prevalence and its social determinants, data from this module were not included in the present analysis.

The determinants of health module (Module 3) covers body weight and height (used to calculate BMI), physical activity (frequency and duration across work, transport, and leisure domains), smoking status, alcohol consumption frequency, and social support. Social support was measured using the Oslo Social Support Scale (OSS-3), which assesses primary support group size, perceived interest from others, and ease of obtaining practical help.

Key social variables collected in addition to the modules include gender, age, country of residence, degree of urbanization, education level, occupation, employment status, marital status, household size and income quintile.

### 2.4. Variables

The dependent variable was pain status, operationalised as a binary variable: respondents reporting any pain (very mild to very severe on the four-week pain intensity scale) were classified as “with pain,” and those reporting no pain were classified as “without pain”.

It should be noted that the EHIS pain measure captures pain experience over the preceding four weeks and does not correspond to established definitions of chronic pain, which typically require a minimum duration of three months. The prevalence estimates reported in this study therefore may reflect recent pain presence rather than chronic pain prevalence, and caution is warranted when comparing these figures with chronic pain prevalence data reported in other studies.

It should be noted that the EHIS contains two conceptually distinct variables involving daily activity limitations. The first, referred to throughout this manuscript as “daily life limitations”, derives from the general health status sub-module (Module 1) and asks respondents whether they have been limited in activities usually performed due to any health problem lasting at least six months; this general functional status indicator was included as an independent variable. The second, referred to as “pain-related limitation in daily activities” or “pain interference”, derives from the dedicated pain chapter within Module 1 and specifically measures the degree to which pain interfered with normal daily activities over the preceding four weeks on a five-category scale (not at all, a little bit, moderately, quite a bit, extremely); this variable is presented descriptively to characterise the burden of pain across countries. Pain interference was not modelled as an independent outcome in the multivariable analyses because it is inherently conditional on reporting pain and is derived from the same pain assessment battery as the primary outcome, which would introduce circularity into the analytical framework.

Independent variables were organised into three domains: (1) sociodemographic characteristics (age, gender, education level, employment status, marital status, household size, income quintile, and degree of urbanization); (2) health status (self-rated health, long-standing health problem, daily life limitations, and the presence of each chronic condition category and accidents/injuries); (3) health determinants (BMI category, physical activity level, daily walking duration, smoking status, alcohol consumption, and social support).

### 2.5. Statistical Analysis

Descriptive statistics were reported as numbers and percentages [n (%)] for categorical variables and as means ± standard deviations (mean ± SD) for continuous variables. Between-country differences were assessed using ANOVA for normally distributed continuous variables, and the Kruskal–Wallis test or chi-square test for ordinal and nominal categorical variables, respectively. All tests were two-sided, and *p*-values below 0.05 were considered statistically significant. Given the large sample sizes in the descriptive analyses, statistical significance should be interpreted alongside the magnitude of between-country differences. All statistically significant between-country comparisons are accompanied by the corresponding percentages or means to allow readers to assess their practical relevance.

Descriptive estimates of pain prevalence were calculated using the EHIS final individual survey weights (variable WGT), which account for each respondent’s probability of selection, correction for unit non-response, and calibration to the distribution of persons in the target population by sex and ten-year age groups, in accordance with the minimum calibration requirements specified in the EHIS methodological manual [21]. All multivariable logistic regression analyses (LASSO variable selection and second-stage standard logistic regression, Section 2.5 and Section 3.6) were performed on unweighted data. This approach was adopted because the analytical goal of the regression was to identify factors independently associated with pain and estimate odds ratios rather than to produce population-representative regression coefficients.

To identify factors independently associated with pain within each country, we performed logistic regression using a two-stage modelling approach. Variables with 10% or more missing data were excluded. The number and percentage of missing observations for each variable by country are provided in Supplementary Table S1; variables that were unavailable or not modelled in a particular country are identified accordingly in Supplementary Table S2. Variables for inclusion in the multivariable models were selected using least absolute shrinkage and selection operator (LASSO) regularisation. LASSO logistic regression was applied separately for each country using all prespecified variables available for that country. Binary variables were coded as 0 (No) and 1 (Yes), ordinal categorical variables were coded using consecutive integer values reflecting the ordering of their categories, and nominal variables with more than two categories were represented by indicator variables with the corresponding reference category. No separate manual standardization was performed before model fitting, as factors were standardized internally by the LASSO implementation. The optimal penalty parameter was determined by 10-fold cross-validation, and the value of λ minimizing the cross-validated prediction error (lambda.min) was selected. Variables with non-zero coefficients at lambda.min were retained and subsequently entered into a standard multivariable binary logistic regression model fitted separately for each country. Adjusted odds ratios (ORs), 95% confidence intervals (95% CIs), and *p*-values were obtained from these post-selection logistic regression models. The variables considered in each country-specific LASSO model and their corresponding coefficients are presented in Supplementary Table S3. Model performance was assessed using Nagelkerke’s R^2^ and the area under the receiver operating characteristic curve (AUC). All analyses were performed using IBM SPSS Statistics version 31 (IBM Corporation, Armonk, NY, USA) and the R programming environment (R Core Team, 2025).

## 3. Results

### 3.1. Sociodemographic Characteristics of the Study Sample by Country

A total of 34,882 respondents from four countries were included in the study: Serbia (RS, n = 13,178), Bulgaria (BG, n = 7540), Norway (NO, n = 7913) and Finland (FI, n = 6251) (Table 1). Statistically significant differences were observed across all examined sociodemographic variables (all *p* < 0.001). The mean age was highest in Finland (55.1 ± 19.0 years) and lowest in Norway (48.4 ± 18.8 years), with Bulgaria (53.5 ± 19.1 years) and Serbia (50.7 ± 19.1 years) intermediate. In all four countries, the largest age group was the 35–64 year category. Female respondents outnumbered male respondents in Serbia (51.2%), Bulgaria (53.9%) and Finland (57.2%); Norway was the only country with a slight male majority (50.2% male vs. 49.8% female).

Educational attainment differed markedly between countries: high education was reported by 39.9% of Finnish and 39.7% of Norwegian respondents, compared with only 21.4% in Bulgaria and 17.9% in Serbia. Employment rates were highest in Norway (60.9%) and lowest in Serbia (35.6%), with unemployment substantially more prevalent in Serbia (18.0%) than in the other three countries (2.0–8.6%). Regarding marital status, married respondents were the most common category in all four countries (Norway 45.4%, Bulgaria 51.1%, Finland 50.0%, and Serbia 59.7%). Household composition showed a strong country gradient: three-or-more-person households were most prevalent in Serbia (71.2%) and least prevalent in Finland (25.6%). Income distribution was broadly similar across countries, though Serbia had the highest proportion in the lowest quintile (20.6%) and Bulgaria the highest proportion in the highest quintile (21.3%). The degree of urbanization differed significantly among Bulgaria, Norway and Finland (data not collected in Serbia; *p* < 0.001): Bulgaria had the highest proportion of urban-dwelling respondents (43.3%), while Norway had the highest proportion in rural areas (35.3%).

### 3.2. Health Status by Country

Self-rated health differed significantly across countries (*p* < 0.001) (Table 2). The most favourable profile was reported in Norway, where 79.0% of respondents rated their health as very good or good and only 7.1% as bad or very bad. Finland had the least favourable self-rated health profile, with only 58.4% rating their health as very good or good and 34.2% rating it as fair. Serbia (63.9%) and Bulgaria (63.4%) showed similar distributions. The prevalence of a long-standing health problem ranged from 39.2% in Norway to 53.3% in Finland (*p* < 0.001). Serious limitations in daily activities were least frequent in Norway (6.3%) and most frequent in Serbia (8.6%).

Among specific chronic conditions, musculoskeletal diseases were the most prevalent and showed the greatest cross-national variation, affecting 54.3% of respondents in Finland more than twice the rate in Norway (28.2%), Serbia (23.7%) and Bulgaria (17.6%) (*p* < 0.001). Cardiovascular disease was most prevalent in Bulgaria (38.1%) and least in Norway (17.4%), while accidents and injuries were most commonly reported in Finland (12.5%) and least in Bulgaria (2.5%) (all *p* < 0.001) (Table 2).

### 3.3. Health Determinants by Country

Regarding health determinants (Table 3), BMI distribution differed significantly across countries (*p* < 0.001), with obesity most prevalent in Finland (21.2%) and least in Bulgaria (14.0%). Physical inactivity, defined as no physical activity beyond walking, was reported by 83.1% of Bulgarian and 69.1% of Serbian respondents, compared with only 23.2% in Norway and 33.3% in Finland. Daily walking of ≥30 min was most commonly reported in Serbia (66.3%) and least in Norway (54.9%). Smoking prevalence was approximately twice as high in Bulgaria (33.6%) and Serbia (31.1%) as in Norway (17.5%) and Finland (14.4%) (*p* < 0.001).

Conversely, binge drinking was most frequently reported in Norway (68.0%) and least in Serbia (35.1%); this variable was not assessed in Finland (*p* < 0.001 for three-country comparison). Social support was most accessible in Serbia, where 67.2% of respondents reported obtaining support “very easily” or “easily,” compared with only 35.5% in Finland (*p* < 0.001).

### 3.4. Pain Prevalence, Intensity and Activity Limitations by Country

Population-level pain prevalence estimates varied markedly across Serbia (35.4%), Bulgaria (41.5%), Norway (43.6%), and Finland (69.9%) (Table 4).

The distribution of pain intensity over the preceding four weeks also differed significantly (*p* < 0.001): moderate, severe or very severe pain was reported by 29.1% of respondents in Finland, 25.8% in Norway, 17.1% in Bulgaria and 15.8% in Serbia. Pain-related limitations in daily activities followed a corresponding gradient: moderate to extreme limitation was reported by 33.6% of respondents in Finland, compared with 16.3% in Serbia, 15.8% in Bulgaria and 13.6% in Norway (*p* < 0.001).

### 3.5. Prevalence of Pain Across Sociodemographic and Health Subgroups

For the pooled four-country sample (N = 33,384 respondents with complete data on the pain outcome variable), the proportion of respondents reporting pain was examined within each subgroup of every sociodemographic, health-status and behavioural variable (Supplementary Table S2). Pain prevalence increased steadily with age, from 30.4% among respondents under 35 years to 45.3% among those aged 35–64 years and 59.5% among those aged 65 years and older (*p* < 0.001). Women reported pain more frequently than men (52.6% vs. 39.0%; *p* < 0.001). The relationship with education level was non-monotonic: pain prevalence was highest among respondents with low education (53.1%), intermediate among those with high education (46.2%), and lowest among those with middle education (42.8%) (*p* < 0.001).

Labour status showed a marked gradient, with the highest pain prevalence among retired respondents (59.2%), followed by those in other labour categories (43.7%) and employed respondents (40.6%); unemployed respondents had the lowest prevalence (33.3%) (*p* < 0.001). A similar age-related pattern was observed for marital status, with widowed respondents showing the highest pain prevalence (64.1%), followed by divorced (50.5%), married (46.7%) and never-married respondents (37.8%; *p* < 0.001). Pain prevalence decreased with increasing household size, from 56.6% among respondents living alone to 38.0% among those in households of three or more (*p* < 0.001), and decreased progressively across income quintiles from 49.8% in the lowest to 40.8% in the highest (*p* < 0.001). Degree of urbanization was also significantly associated with pain, with the highest prevalence in rural areas (53.7%; *p* = 0.002).

Health-status indicators showed the strongest and most consistent gradients. Pain prevalence rose from 33.1% among respondents rating their health as very good or good to 83.5% among those rating it as bad or very bad (*p* < 0.001), and from 35.7% among those with no daily life limitations to 82.8% among those with serious limitations (*p* < 0.001). Respondents with a long-standing health problem reported pain nearly twice as often as those without one (65.1% vs. 30.1%; *p* < 0.001). Among specific chronic conditions, pain was most prevalent among respondents with musculoskeletal disorders (77.2% vs. 33.0% without; *p* < 0.001), followed by genitourinary disease (76.1%), mental health disorders (75.8%), respiratory disease (67.2%), accidents/injuries (66.3%), endocrine/metabolic disorders (63.9%) and cardiovascular disease (61.8%) (all *p* < 0.001).

Behavioural determinants showed smaller but statistically significant gradients. Pain prevalence increased with BMI category, from 41.3% among respondents of normal weight to 56.1% among those with obesity (*p* < 0.001), and was higher among those reporting less daily walking (51.0% vs. 41.0% for <30 vs. ≥30 min/day; *p* < 0.001). Counterintuitively, pain prevalence was lower among current smokers (43.9% vs. 48.8%) and among those reporting binge drinking (39.3% vs. 45.1%) than their respective reference groups (both *p* < 0.001) (Supplementary Table S2). Pain prevalence increased steadily as perceived social support became harder to obtain, from 40.3% among those for whom support was very easy to obtain to 62.2% among those for whom it was very difficult (*p* < 0.001).

### 3.6. Factors Associated with Pain: Country-Specific Logistic Regression Models

**Figure 1:**
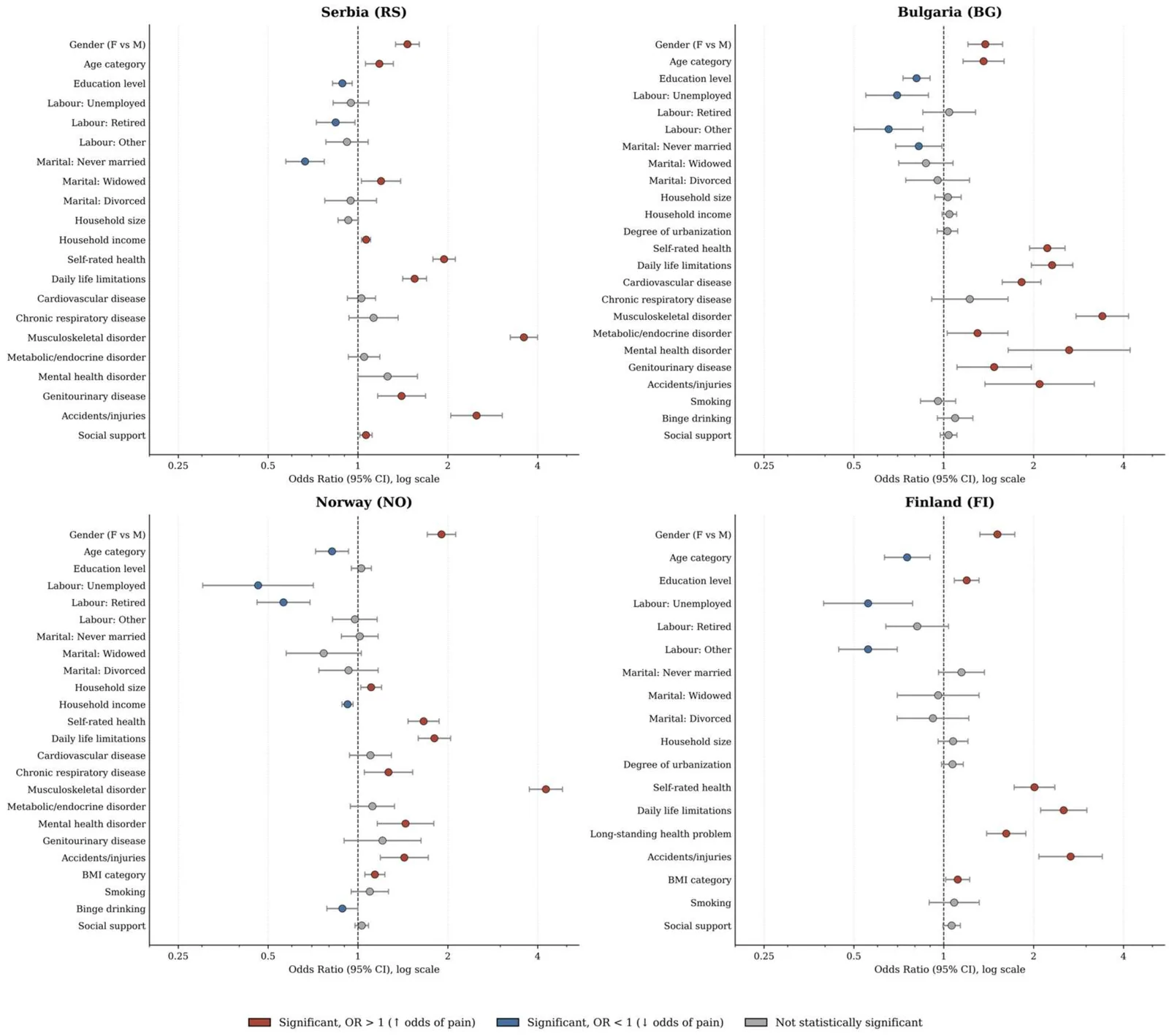
Forest plots of adjusted odds ratios for the presence of pain, by country. Points show adjusted odds ratios (OR) with 95% confidence intervals (horizontal lines) on a logarithmic *x*-axis; the dashed vertical line marks OR = 1 (no association). Red = statistically significant association with increased odds of pain; blue = statistically significant association with decreased odds of pain; grey = not statistically significant (95% CI crosses 1). RS = Serbia, BG = Bulgaria, NO = Norway, FI = Finland.

To identify factors independently associated with pain in each country while managing collinearity among sociodemographic variables (all variance inflation factors < 5.0), LASSO logistic regression was applied separately in each country to select the most informative factors associated with pain (Supplementary Table S2). The LASSO-retained factors were subsequently entered into a standard binary logistic regression model in each country, yielding adjusted odds ratios (OR) with 95% confidence intervals. Table 5 presents the significant factors associated with pain for each country; Supplementary Tables S4–S7 present the full model output; and Figure 1 displays all odds ratios graphically as country-specific forest plots. Model discrimination was good to very good across all countries (AUC range 0.742–0.839), with the Bulgarian model showing the strongest discrimination (AUC = 0.839, Nagelkerke R^2^ = 0.438) and the Finnish model the weakest (AUC = 0.742, Nagelkerke R^2^ = 0.208).

In Serbia (Supplementary Table S4; n = 12,124), the musculoskeletal disorder showed the largest OR among significant factors (OR = 3.60, 95% CI 3.24–4.00, *p* < 0.001), followed by accidents/injuries (OR = 2.50), poorer self-rated health (OR = 1.94), greater daily life limitations (OR = 1.55), female gender (OR = 1.47), genitourinary disease (OR = 1.40), older age category (OR = 1.18), higher household income (OR = 1.07) and greater social support (OR = 1.06). Retirement (OR = 0.84) and never-married status relative to married (OR = 0.67) were associated with lower odds of pain.

In Bulgaria (Supplementary Table S5; n = 6734), the model showed the strongest overall discrimination. Musculoskeletal disorder (OR = 3.40, *p* < 0.001), mental health disorder (OR = 2.63), daily life limitations (OR = 2.31), self-rated health (OR = 2.22) and accidents/injuries (OR = 2.10) showed the five strongest associations with pain. Cardiovascular disease (OR = 1.82), genitourinary disease (OR = 1.50), metabolic/endocrine disorder (OR = 1.30), older age category (OR = 1.36) and female gender (OR = 1.38) were also significant. Unemployment (OR = 0.70), other labour status (OR = 0.65), and higher education (OR = 0.81, i.e., each step-up in education was associated with lower odds) were associated with lower odds of pain.

In Norway (Supplementary Table S6; n = 7236), musculoskeletal disorder showed the largest effect size of any factor (OR = 4.26, 95% CI 3.75–4.85, *p* < 0.001). Female gender (OR = 1.91), daily life limitations (OR = 1.80), self-rated health (OR = 1.66), mental health disorder (OR = 1.44), accidents/injuries (OR = 1.43), chronic respiratory disease (OR = 1.27), BMI category (OR = 1.14), and larger household size (OR = 1.11) were also significantly associated with higher odds of pain. Unemployment (OR = 0.46) and retirement (OR = 0.56) were associated with markedly lower odds of pain, a direction discussed further in Section 4. Higher household income (OR = 0.92) and older age category (OR = 0.82) were also associated with lower odds.

In Finland (Supplementary Table S7; n = 5193), the LASSO procedure operated on a smaller candidate set than in the other three countries, because all chronic disease category variables in the Finnish EHIS microdata had missing data rates exceeding the pre-specified 10% exclusion threshold (musculoskeletal disorder 14.9%, cardiovascular disease 16.6%, chronic respiratory disease 16.2%, endocrine/metabolic disorders 15.3%, mental health disorders 14.6%, and genitourinary diseases 15.0%; see Supplementary Table S1) and were therefore excluded from Finland’s candidate set before LASSO was applied. A long-standing health problem, which captures a broad summary of chronic health burden and has an acceptable rate of missing data, was retained as an alternative health-status indicator. Accidents/injuries (OR = 2.66), daily life limitations (OR = 2.52), self-rated health (OR = 2.01) and long-standing health problems (OR = 1.62) showed the strongest associations with pain. Female gender (OR = 1.53), higher education (OR = 1.19) and BMI category (OR = 1.11) were also significant. Unemployment (OR = 0.56) and other labour statuses (OR = 0.55) were associated with lower odds of pain.

Across all four countries (Figure 1), accidents/injuries had a consistent positive association with pain (OR range 1.43–2.66), while musculoskeletal disorder consistently showed the strongest association with pain where retained (OR range 3.40–4.26), together with poorer self-rated health (OR range 1.66–2.22), greater daily life limitations (OR range 1.55–2.52) and female gender (OR range 1.38–1.91). In contrast, the direction and significance of labour status, marital status, household composition, income, education and behavioural determinants varied across countries, indicating that structural and contextual factors are associated with pain in ways that are country-specific.

## 4. Discussion

This multinational study demonstrates that pain prevalence varies substantially across four European countries with contrasting socioeconomic and health system contexts, ranging from 35.4% in Serbia to 69.9% in Finland, and that this variation is systematically associated with a broad set of social determinants of health. The analyses identified two conceptually distinct groups of factors associated with pain. Clinical and health-status factors, including musculoskeletal disorders, accidents/injuries, self-rated health, and daily life limitations, showed some of the largest and most consistent associations when these variables were available for analysis, which is expected given their conceptual and biological proximity to the pain outcome. Social determinants in the upstream sense (including income, education, employment, household composition and social support) showed smaller but policy-relevant associations that varied meaningfully by national context, underscoring that the structural conditions in which people live contribute to differential pain burden in ways that extend beyond clinical morbidity. These findings extend prior evidence from single-country studies and contribute cross-national comparative data that are largely absent from the existing literature, particularly for Serbia and Bulgaria. Although data on the prevalence and social determinants of pain in the general populations of Serbia and Bulgaria are limited, previous studies conducted in specific population groups, including adolescents, individuals aged 65 years and older, and people living with chronic conditions, indicate associations between various social determinants and the occurrence and characteristics of pain [22,23].

The wide cross-national variation in pain prevalence observed in our study is consistent with prior population-based research. Zimmer et al., using World Health Survey data from 52 countries, estimated pain prevalence between 9.9% and 50.3% among adults aged 25 years and older [24], while European meta-analyses have reported prevalence rates of 12–48% with marked regional heterogeneity [14]. Our estimates fall within and extend this range, with Finland’s prevalence of 69.9% among the highest reported in a general population European survey, likely reflecting the high burden of musculoskeletal disease in that sample (54.3%), combined with Finland’s older age structure and greater reporting openness in Nordic health survey contexts. The exclusion of musculoskeletal disorders from Finland’s model should therefore be understood primarily as a consequence of data availability constraints rather than of its epidemiological relationship with pain in that country. Whether a musculoskeletal disorder would have been retained in the Finnish model had complete data been available cannot be established from the present analyses; its relationship with other health-status variables and the behaviour of penalised regression in the presence of correlated factors may also have played a role. By contrast, Serbia’s lower prevalence (35.4%) may reflect both a younger sample distribution and differences in pain reporting in settings where pain-related help-seeking is less normalised. Insufficient recognition of pain and limited access to multidisciplinary care are known to contribute to socially patterned differences in pain outcomes [25], a mechanism particularly relevant to the Serbian and Bulgarian contexts.

Across all four countries where the variable was available for analysis, musculoskeletal disorder showed the largest association with pain (OR range 3.39–4.26 in Serbia, Bulgaria and Norway), followed by poorer self-rated health (OR range 1.66–2.23 across all four countries) and greater daily life limitations (OR range 1.55–2.56 across all four countries). In Finland, musculoskeletal disorder and all other chronic disease category variables were excluded from the model due to missing data exceeding the pre-specified 10% threshold (Supplementary Table S1); long-standing health problem was retained as an alternative summary indicator of chronic health burden (OR 1.62). The large magnitude of these clinical health-status associations is not surprising given that musculoskeletal disorders are in many cases concurrent conditions of pain, and that self-rated health and daily life limitations partially reflect pain experience itself. These variables should therefore be understood as proximal clinical correlates of pain rather than upstream social determinants in the structural sense. Female gender was also consistently associated with pain across all four countries (OR range 1.38–1.91), occupying an intermediate position as both a biological characteristic and a social category that presents structural inequalities in health. This finding is consistent with a large body of literature documenting the central role of musculoskeletal conditions in population-level pain burden [26], and aligns with EHIS-based analyses showing that musculoskeletal diseases are among the most prevalent chronic conditions in Europe. The consistency of this association across the countries in which musculoskeletal disorder was included in the model is consistent with a substantial contribution of musculoskeletal morbidity to population-level pain burden, although the present analyses cannot establish its causal role or determine whether its association would have remained significant in Finland under complete data availability. Female gender was also significantly associated with pain in all four models (OR range 1.38–1.91), consistent with evidence of a higher chronic pain burden among women across Europe, attributed to a combination of biological, structural, cultural and healthcare access factors [27]. Poorer self-rated health and greater limitations in daily activities were similarly associated with pain across all countries, corroborating findings from quality-of-life studies showing bidirectional relationships between pain, functional limitation and self-perceived health status [28,29].

Turning to upstream social determinants (income, education, employment, household composition, degree of urbanization and social support), the associations with pain were smaller in magnitude than those of clinical health-status factors but carried important public health significance for two reasons. First, they are potentially modifiable through policy intervention in ways that clinical morbidity is not. Second, their cross-national variation reveals the degree to which structural conditions specific to each country’s socioeconomic and health system context shape the distribution of pain in the population. Beyond these significant associations, the associations of sociodemographic and behavioural determinants with pain showed marked cross-national variation, which is among the most substantively important findings of this study. The sociodemographic gradient in pain, with higher prevalence among older, less educated, widowed, and lower-income respondents, was more pronounced in Serbia and Bulgaria than in Nordic countries. Serbia and Bulgaria are characterised by greater socioeconomic inequality, less comprehensive social protection systems, and higher out-of-pocket health expenditure [7,25,28], and these contextual differences offer a plausible explanatory frame for the observed gradient. However, as these country-level characteristics were not directly measured or modelled, the attribution of steeper socioeconomic pain gradients specifically to social protection system differences cannot be established from the current data alone. These patterns are consistent with evidence that lower socioeconomic status is associated with increased risk of chronic pain conditions including low back pain [8], and with Rometsch et al.’s finding that female gender, older age, absence of a partner relationship, lower education and unemployment are independently associated with chronic pain in European populations [14]. The inverse associations between unemployment and pain observed in Bulgaria, Norway and Finland, while counterintuitive, may partly reflect age selection: unemployed respondents in these countries tend to be substantially younger than employed or retired respondents, and younger age is strongly associated with lower pain prevalence in this sample. The cross-sectional design prevents determination of the temporal direction of this relationship, and we present this observation as a finding warranting further investigation rather than as an established mechanism. Similar associations have also been confirmed in population-based cohorts, including the HUNT Study, in which demographic, socioeconomic, and risk factors were associated with the presence of pain [30]. In Norway, previous research further indicates a complex relationship between economic status, social participation, and pain: long-term recipients of social assistance who experience chronic pain have significantly poorer physical and mental health compared with those without pain, suggesting a mutually reinforcing relationship between economic disadvantage, social isolation, and pain [31]. In Finland, educational attainment and occupational status have also been associated with the prevalence of back and joint pain [32]. In contrast, in Norway and Finland, the association of employment with pain and the comparatively weaker gradient across income quintiles may be consistent with a buffering association of comprehensive social security networks and universal healthcare coverage on the relationship between socioeconomic disadvantage and pain, an interpretation that is theoretically plausible and supported by the broader Nordic welfare state literature [15,16]. However, since country-level policy characteristics were not directly measured or modelled in our analyses, this remains a contextual hypothesis rather than a demonstrated mechanism. The observed differences between country groups could equally reflect differences in population age structure, chronic disease profiles, survey implementation practices, or cultural norms around pain reporting that are unmeasured in the current dataset.

The association between pain and specific chronic conditions also showed country-specific patterns. Cardiovascular disease, mental health disorders and genitourinary disease contributed significantly to the Bulgarian model but showed comparatively weaker associations in Serbia, possibly reflecting differences in the prevalence of multimorbidity and in health system capacity to identify and treat these conditions. Our finding of a strong association between mental health disorders and pain in Bulgaria (OR 2.63) and Norway (OR 1.44) aligns with evidence that depression and insomnia are significantly associated with pain in multiple anatomical locations [33], and with meta-analytic data documenting high pain prevalence across psychiatric conditions. The absence of a significant association between chronic kidney disease and pain in our sample, in contrast to the near-60% prevalence reported by Davison et al. [34], likely reflects the low overall prevalence of this condition in our general population sample and the consequent limited statistical power to detect this association.

Regarding behavioural determinants, the counterintuitive finding that binge drinking was associated with lower odds of pain in the pooled sample warrants careful interpretation. Both binge drinking and smoking were more common among younger respondents in our sample, and the apparent association is most plausibly explained by age confounding once age is accounted for in the multivariable models; the direction of the smoking association was attenuated or reversed in several country-specific models, but was not significant. This interpretation is consistent with the literature and underscores the importance of adjusted analyses in cross-sectional surveys with wide age ranges [35]. By contrast, higher BMI was associated with significantly increased odds of pain in Norway and Finland, consistent with a systematic review and meta-analysis by Qian et al. showing that overweight and obesity are associated with chronic pain in older populations [36]. The absence of a significant BMI-pain association in Serbia and Bulgaria may reflect the younger age distribution of those samples or threshold associations in pain reporting. Poorer social support was associated with greater pain prevalence, consistent with Rinaudo et al.’s finding that lower social support is associated with worse pain outcomes [37], though the strength and significance of this association varied by country, suggesting that the role of social networks is itself affected by the broader social environment.

An important interpretive limitation of the present study is that, while we identified a broad set of social, health-status and behavioral correlates of pain, our analyzes were unable to capture the psychological mechanisms through which these factors may translate into pain burden and interference. The EHIS does not include measures of psychological constructs such as pain catastrophizing, pain self-efficacy or emotional regulation, which may contribute to variation in pain and functional interference [12,13]. Pain catastrophizing in particular, characterized by rumination, magnification and perceived helplessness in the face of pain, has been shown to amplify the relationship between chronic conditions such as musculoskeletal disease and both pain and functional interference [12,13]. The strong associations we observed between pain and daily life limitations across all four countries may therefore reflect not only the direct functional burden of pain but also the amplifying role of psychological processes that operate alongside structural and clinical determinants. Future population-based surveys incorporating validated measures of pain-related psychological constructs would allow a more complete modelling of the pathways from social conditions to pain outcomes.

Several limitations of this study should be acknowledged. First, the cross-sectional design permits identification of associations between social determinants and pain but does not allow causal inference or determination of the temporal sequence of these relationships. The binary pain outcome, combining all levels of pain severity from very mild to very severe, produces a heterogeneous outcome category that may contribute to the high prevalence observed in Finland and reduces sensitivity to clinically meaningful differences in pain severity. The pain-related limitation in daily activities variable, a five-category measure of pain interference, was presented descriptively but not modelled as a multivariable outcome, as doing so would require a separate analysis restricted to respondents reporting pain, constituting a distinct research question. Future analyses using ordinal or threshold-based outcome definitions would allow more refined examination of the social determinants of pain intensity specifically. Also, future studies examining social determinants of pain interference specifically, among those already experiencing pain, would complement the prevalence-focused approach adopted here and provide additional insight into the factors associated with pain burden beyond mere presence. Second, the two-stage modelling approach in which LASSO was used for variable selection and standard logistic regression was subsequently fitted on the retained factors associated with pain does not fully correct for the multiple testing implications of data-driven selection, and *p*-values from the second-stage models should be interpreted as descriptive of relative association strength rather than as confirmatory inferential tests. Third, although the EHIS uses a harmonised instrument, country-specific differences in translation, cultural interpretation of pain, and health literacy may introduce unmeasured measurement heterogeneity. Fourth, sample sizes differed between countries, and a degree of missing data was present for certain variables, particularly binge drinking in Finland, which was not assessed. Fifth, temporal misalignment between outcome and factor reference periods, pain assessed over the preceding four weeks versus chronic conditions assessed over the preceding twelve months, represents a potential source of measurement inconsistency. All variables in this study, including the primary outcome, were based on self-reported data. Self-report bias may affect the accuracy of pain assessment as well as the reporting of chronic conditions, BMI, physical activity, alcohol consumption and socioeconomic variables, particularly in contexts where social desirability, health literacy or recall accuracy varies systematically across countries or population subgroups. Such bias may differentially affect prevalence estimates and the magnitude of observed associations across the four countries.

Although the regression models included a comprehensive set of social, health-status and behavioural variables, residual confounding cannot be excluded. Variables not available in the EHIS, including psychological characteristics, built environment features, occupational exposures, and pain history, may confound the associations reported, and their absence means that the observed odds ratios should be interpreted as partially adjusted estimates rather than as measures of independent association. The study population was aged 15 years and older, combining adolescents, working-age adults and older adults whose social determinants of pain, pain mechanisms, and the meaning of pain may differ substantially across the life course. Age was included as a covariate in all regression models, but statistical adjustment for age alone may not fully capture the heterogeneity in pain determinants across these distinct life stages. Future analyses stratified by broad age group would allow more targeted examination of social determinants of pain within each life course phase.

The four-country design does not allow disentanglement of country-level policy effects from other sources of between-country heterogeneity, including differences in population composition, age structure, health profiles, pain reporting behaviour, cultural norms around pain expression, and survey implementation procedures [38]. Country-level characteristics such as welfare state model and healthcare system organisation were not directly measured or included as variables in the analyses. Cross-national differences in associations should therefore be interpreted as descriptive findings that are consistent with contextual explanations, rather than as evidence of specific policy or system-level mechanisms. Multilevel modelling incorporating country-level macro-indicators, such as Gini coefficients, healthcare expenditure per capita, or universal health coverage indices, across a larger number of countries would be needed to test these hypotheses more rigorously [24,39].

This study has several notable strengths. The study is based on nationally representative probability samples in all four countries, drawn from census and population registers using country-specific probability sampling designs, ensuring that findings are generalisable to the non-institutionalised population aged 15 years and older in each country—a level of representativeness that clinical and convenience samples cannot achieve. The country-specific modelling strategy, fitting separate logistic regression models for each country rather than relying exclusively on a pooled analysis, preserves country-level heterogeneity and allows identification of context-specific patterns of association that would be obscured in a single aggregated model. The study is among the few cross-national pain analyses to include Serbia and Bulgaria alongside Nordic countries, addressing a significant geographic and socioeconomic gap in the European pain literature, where evidence from Southeast European transitional health systems is substantially underrepresented. Finally, the breadth of the determinant framework, simultaneously examining sociodemographic, clinical health-status and behavioural variables across three conceptual domains, provides a more integrative picture of the social patterning of pain than studies focusing on a single determinant domain, and the use of publicly available Eurostat microdata ensures that the analytical approach is transparent and potentially replicable by other research teams who obtain data access.

The use of the harmonised EHIS instrument across all four countries ensures a high degree of cross-national comparability of both outcome and social determinant measurements, which is rarely achievable in multinational pain research. The large combined sample (n = 34,882) provides reasonable statistical power to detect associations of modest magnitude in country-specific models, though the effective sample size under the complex survey design may differ from the nominal sample, and power varies across country-specific analyses given their substantially different sample sizes. The deliberate selection of countries representing contrasting welfare state models and socioeconomic development stages, Nordic high-income countries alongside Southeast European transitional economies, maximises variability in the social determinants examined and strengthens the capacity to distinguish context-specific factors associated with pain. Finally, the LASSO-based variable selection approach was used to promote parsimony and reduce the risk of overfitting that characterises conventional stepwise regression in large predictor sets; formal reproducibility of the selected models through external or repeated internal validation was not assessed and remains a direction for future work.

## 5. Conclusions

This multinational analysis provides evidence that pain prevalence is substantially associated with social determinants of health, and that the magnitude and direction of these associations vary meaningfully by national context. Clinical health-status factors, musculoskeletal disorders (in Serbia, Bulgaria and Norway, where data were available), accidents/injuries, poorer self-rated health, long-standing health problems and limitations in daily activities were the strongest correlates of pain across the country-specific models and may reflect their proximal relationship with the pain outcome. Upstream social determinants, including education, employment, income, household composition and social support, showed smaller but policy-relevant associations that varied by national context in ways consistent with differences in socioeconomic conditions and health system organisation, though not causally attributable to these given the cross-sectional design. The female gender was consistently associated with pain across all four countries as both a biological and social determinant of health. These findings confirm that clinical morbidity represents the strongest correlate of pain at the population level, and that effective pain prevention requires addressing these clinical conditions directly. At the same time, the country-level associations of upstream social determinants suggest that structural conditions (employment, income, education, and social support) are associated with pain burden and should be incorporated into comprehensive public health approaches to pain that complement clinical management and are tailored to the socioeconomic realities of each national context. Effective pain management should also integrate assessment of psychological processes, including pain catastrophizing, which has been shown to amplify the relationship between musculoskeletal conditions and both pain intensity and functional interference, alongside biological and social evaluation, which may reflect a genuinely biopsychosocial approach to pain that extends beyond structural intervention alone.

## Figures and Tables

**Table 1:** Sociodemographic characteristics of respondents by country.

Variables	RS n = 13,178	BG n = 7540	NO n = 7913	FI n = 6251	Statistical Test, Overall *p*
Age, mean ± SD	50.7 ± 19.1	53.5 ± 19.1	48.4 ± 18.8	55.1 ± 19.0	*p* < 0.001 *
Age group, n (%)					
<35 years	3148 (23.9%)	1428 (18.9%)	2193 (27.7%)	1141 (18.3%)	*p* < 0.001 **
35–64 years	6325 (48.0%)	3606 (47.8%)	3895 (49.2%)	2699 (43.2%)	
≥65 years	3705 (28.1%)	2506 (33.2%)	1825 (23.1%)	2411 (38.6%)	
Gender, n (%)					
Male	6431 (48.8%)	3477 (46.1%)	3973 (50.2%)	2676 (42.8%)	*p* < 0.001 ***
Female	6747 (51.2%)	4063 (53.9%)	3940 (49.8%)	3575 (57.2%)	
Education level, n (%)					
Low	3525 (26.8%)	2074 (27.5%)	1603 (21.0%)	1437 (23.0%)	*p* < 0.001 **
Middle	7289 (55.3%)	3854 (51.1%)	2992 (39.2%)	2317 (37.1%)	
High	2356 (17.9%)	1610 (21.4%)	3030 (39.7%)	2497 (39.9%)	
Labour status, n (%)					
Employed	4687 (35.6%)	3405 (45.2%)	4794 (60.9%)	2600 (42.2%)	*p* < 0.001 ***
Unemployed	2365 (18.0%)	649 (8.6%)	156 (2.0%)	251 (4.1%)	
Retired	4183 (31.8%)	2695 (35.8%)	1582 (20.1%)	2454 (39.8%)	
Other	1921 (14.6%)	787 (10.4%)	1343 (17.1%)	860 (13.9%)	
Marital status, n (%)					
Never married	2966 (22.6%)	1945 (25.9%)	3359 (42.5%)	2116 (34.2%)	*p* < 0.001 ***
Married	7856 (59.7%)	3847 (51.1%)	3585 (45.4%)	3096 (50.0%)	
Widowed	1672 (12.7%)	1247 (16.6%)	392 (5.0%)	470 (7.6%)	
Divorced	658 (5.0%)	484 (6.4%)	569 (7.2%)	505 (8.2%)	
Household size, n (%)					
One	1013 (7.7%)	1228 (16.3%)	1978 (25.0%)	1615 (26.1%)	*p* < 0.001 **
Two	2782 (21.1%)	2286 (30.3%)	2879 (36.4%)	2989 (48.3%)	
Three and more	9383 (71.2%)	4026 (53.4%)	3056 (38.6%)	1585 (25.6%)	
Income quintile, n (%)					
1st quintile	2718 (20.6%)	1145 (15.8%)	1446 (18.3%)	1108 (20.0%)	*p* < 0.001 **
2nd quintile	2695 (20.5%)	1325 (18.2%)	1555 (19.7%)	1107 (20.0%)	
3rd quintile	2662 (20.2%)	1611 (22.2%)	1592 (20.1%)	1107 (20.0%)	
4th quintile	2640 (20.0%)	1634 (22.5%)	1676 (21.2%)	1108 (20.0%)	
5th quintile	2463 (18.7%)	1548 (21.3%)	1637 (20.7%)	1108 (20.0%)	
Degree of urbanization, n (%)					
Cities	NA	3266 (43.3%)	1853 (23.4%)	2431 (38.9%)	*p* < 0.001 ***
Suburbs	NA	2485 (33.0%)	3269 (41.3%)	2099 (33.6%)	
Rural areas	NA	1789 (23.7%)	2791 (35.3%)	1721 (27.5%)	

RS = Serbia, BG = Bulgaria, NO = Norway, FI = Finland. NA = not available/not assessed in that country. * ANOVA; ** Kruskal–Wallis; *** Chi-square.

**Table 2:** Health status of respondents by country.

Variables	RS n = 13,178	BG n = 7540	NO n = 7913	FI n = 6251	Statistical Test, Overall *p*
Self-rated health, n (%)					
Very good and good	7966 (63.9%)	4587 (63.4%)	6249 (79.0%)	3420 (58.4%)	*p* < 0.001 *
Fair	3041 (24.4%)	1790 (24.8%)	1095 (13.8%)	2005 (34.2%)	
Bad and very bad	1453 (11.7%)	854 (11.8%)	564 (7.1%)	432 (7.4%)	
Long-standing health problem, n (%)					
Yes	6050 (46.0%)	3496 (46.4%)	3100 (39.2%)	3277 (53.3%)	*p* < 0.001 **
No	7116 (54.0%)	4038 (53.6%)	4811 (60.8%)	2873 (46.7%)	
Daily life limitations, n (%)					
Not limited	10,233 (77.7%)	5666 (75.2%)	6425 (81.4%)	3861 (63.7%)	*p* < 0.001 *
Limited	1806 (13.7%)	1343 (17.8%)	971 (12.3%)	1828 (30.2%)	
Seriously limited	1126 (8.6%)	527 (7.0%)	500 (6.3%)	372 (6.1%)	
Cardiovascular diseases, n (%)					
Yes	4659 (35.4%)	2866 (38.1%)	1379 (17.4%)	1586 (30.4%)	*p* < 0.001 **
No	8493 (64.6%)	4651 (61.9%)	6533 (82.6%)	3629 (69.6%)	
Chronic respiratory diseases, n (%)					
Yes	783 (5.9%)	415 (5.5%)	763 (9.6%)	518 (9.9%)	*p* < 0.001 **
No	12,386 (94.1%)	7111 (94.5%)	7149 (90.4%)	4722 (90.1%)	
Musculoskeletal diseases, n (%)					
Yes	3120 (23.7%)	1317 (17.6%)	2233 (28.2%)	2886 (54.3%)	*p* < 0.001 **
No	10,046 (76.3%)	6181 (82.4%)	5679 (71.8%)	2432 (45.7%)	
Endocrine and metabolic diseases, n (%)					
Yes	2247 (17.1%)	769 (10.4%)	1098 (13.9%)	1538 (29.1%)	*p* < 0.001 **
No	10,864 (82.9%)	6623 (89.6%)	6814 (86.1%)	3756 (70.9%)	
Mental health diseases, n (%)					
Yes	617 (4.7%)	231 (3.1%)	632 (8.0%)	396 (7.4%)	*p* < 0.001 **
No	12,550 (95.3%)	7254 (96.9%)	7280 (92.0%)	4945 (92.6%)	
Genitourinary diseases, n (%)					
Yes	922 (7.0%)	584 (7.8%)	330 (4.2%)	562 (10.6%)	*p* < 0.001 **
No	12,244 (93.0%)	6899 (92.2%)	7582 (95.8%)	4749 (89.4%)	
Accidents and injuries, n (%)					
Yes	671 (5.1%)	189 (2.5%)	697 (8.8%)	746 (12.5%)	*p* < 0.001**
No	12,498 (94.9%)	7350 (97.5%)	7203 (91.2%)	5236 (87.5%)	

RS = Serbia, BG = Bulgaria, NO = Norway, FI = Finland. * Kruskal–Wallis; ** Chi-square.

**Table 3:** Health determinants of respondents by country.

Variables	RS n = 13,178	BG n = 7540	NO n = 7913	FI n = 6251	Statistical Test, Overall *p*
BMI category, n (%)					
Normal weight (<25)	5351 (46.1%)	3142 (43.8%)	3787 (49.0%)	2308 (40.1%)	*p* < 0.001 *
Overweight (25–29.9)	4224 (36.4%)	3029 (42.2%)	2846 (36.9%)	2226 (38.7%)	
Obese (≥30)	2023 (17.4%)	1007 (14.0%)	1089 (14.1%)	1217 (21.2%)	
Physical activity (excl. walking), n (%)					
Never	8608 (69.1%)	6006 (83.1%)	1831 (23.2%)	1807 (33.3%)	*p* < 0.001 *
1–3 days/week	1777 (14.3%)	767 (10.6%)	3285 (41.7%)	2452 (45.2%)	
4–5 days/week	736 (5.9%)	253 (3.5%)	1632 (20.7%)	807 (14.9%)	
6–7 days/week	1332 (10.7%)	201 (2.8%)	1138 (14.4%)	364 (6.7%)	
Daily walking duration, n (%)					
<30 min/day	3866 (33.7%)	2290 (34.3%)	2459 (45.1%)	2035 (39.0%)	*p* < 0.001 **
≥30 min/day	7599 (66.3%)	4388 (65.7%)	2990 (54.9%)	3182 (61.0%)	
Smoking, n (%)					
Yes	2872 (31.1%)	2427 (33.6%)	1383 (17.5%)	878 (14.4%)	*p* < 0.001 **
No	6353 (68.9%)	4790 (66.4%)	6505 (82.5%)	5230 (85.6%)	
Binge drinking, n (%)					
Yes	2979 (35.1%)	3974 (55.1%)	5353 (68.0%)	NA	*p* < 0.001 **
No	5498 (64.9%)	3235 (44.9%)	2523 (32.0%)	NA	
Social support, n (%)					
Very easy	2579 (21.1%)	1091 (15.2%)	2258 (29.3%)	660 (11.5%)	*p* < 0.001 *
Easy	5651 (46.1%)	2557 (35.6%)	3146 (40.8%)	1380 (24.0%)	
Possible	2822 (23.0%)	2649 (36.8%)	1436 (18.6%)	2657 (46.2%)	
Difficult	924 (7.5%)	699 (9.7%)	614 (8.0%)	646 (11.2%)	
Very difficult	272 (2.2%)	195 (2.7%)	263 (3.4%)	414 (7.2%)	

RS = Serbia, BG = Bulgaria, NO = Norway, FI = Finland. NA = not available/not assessed in that country (binge drinking not assessed in Finland). * Kruskal–Wallis; ** Chi-square.

**Table 4:** Pain status, pain intensity and pain-related limitations by country.

Variables	RS n = 13,178	BG n = 7540	NO n = 7913	FI n = 6251	Statistical Test, Overall *p*
Pain status, n (%)					
With pain	4665 (35.4%)	3129 (41.5%)	3450 (43.6%)	4369 (69.9%)	*p* < 0.001 *
Without pain	8513 (64.6%)	4411 (58.5%)	4463 (56.4%)	1882 (30.1%)	
Pain in last 4 weeks, n (%)					
None	7940 (63.7%)	3830 (53.1%)	4466 (56.5%)	1727 (29.7%)	*p* < 0.001 **
Very mild and mild	2552 (20.5%)	2155 (29.9%)	1396 (17.7%)	2445 (42.1%)	
Moderate	1267 (10.2%)	876 (12.1%)	1326 (16.8%)	1176 (20.3%)	
Severe	561 (4.5%)	295 (4.1%)	552 (7.0%)	386 (6.6%)	
Very severe	141 (1.1%)	62 (0.9%)	158 (2.0%)	73 (1.3%)	
Limitation in daily activities due to pain, n (%)					
Not at all	8756 (70.3%)	4664 (64.7%)	5930 (75.1%)	1394 (31.9%)	*p* < 0.001 **
A little bit	1673 (13.4%)	1409 (19.5%)	897 (11.4%)	1511 (34.6%)	
Moderately	1076 (8.6%)	742 (10.3%)	459 (5.8%)	1035 (23.7%)	
Quite a bit	669 (5.4%)	324 (4.5%)	328 (4.2%)	340 (7.8%)	
Extremely	285 (2.3%)	74 (1.0%)	282 (3.6%)	91 (2.1%)	

RS = Serbia, BG = Bulgaria, NO = Norway, FI = Finland. * Chi-square; ** Kruskal–Wallis.

**Table 5:** Models explaining significant factors associated with pain by country.

Significant Factors	OR and 95% CI			
	RS n = 12,124 Nagelkerke R^2^ = 0.344; AUC = 0.802	BG n = 6734 Nagelkerke R^2^ = 0.438; AUC = 0.839	NO n = 7236 Nagelkerke R^2^ = 0.274; AUC = 0.760	FI n = 5193 Nagelkerke R^2^ = 0.208; AUC = 0.742
Gender (female vs. male)	1.465	1.378	1.905	1.513
	(1.337–1.605)	(1.206–1.575)	(1.708–2.125)	(1.322–1.731)
Age category	1.180	1.360	0.819	0.754
	(1.060–1.314)	(1.161–1.593)	(0.721–0.930)	(0.633–0.899)
Education level	0.887	0.811		1.194
	(0.822–0.957)	(0.731–0.900)		(1.085–1.313)
Labour status: Unemployed		0.698	0.463	0.558
		(0.548–0.88)	(0.302–0.709)	(0.396–0.787)
Labour status: Retired	0.842		0.563	
	(0.725–0.978)		(0.459–0.691)	
Labour status: Other		0.654		0.558
		(0.501–0.853)		(0.445–0.699)
Marital status: Never married	0.665	0.825		
	(0.573–0.771)	(0.690–0.986)		
Marital status: Widowed	1.195			
	(1.027–1.390)			
Household size			1.107	
			(1.022–1.200)	
Household income	1.065		0.923	
	(1.028–1.103)		(0.884–0.963)	
Self-rated health	1.944	2.223	1.660	2.014
	(1.783–2.120)	(1.939–2.549)	(1.472–1.871)	(1.722–2.356)
Daily life limitations	1.549	2.307	1.804	2.523
	(1.413–1.698)	(1.966–2.707)	(1.592–2.045)	(2.112–3.015)
Cardiovascular disease		1.8244		
		(1.571–2.118)		
Chronic respiratory disease			1.266	
			(1.051–1.525)	
Musculoskeletal disorder	3.598	3.400	4.265	
	(3.238–3.998)	(2.775–4.165)	(3.752–4.848)	
Metabolic/endocrine disorder		1.298		
		(1.027–1.641)		
Mental health disorder		2.631	1.444	
		(1.644–4.211)	(1.161–1.795)	
Genitourinary disease	1.400	1.476		
	(1.164–1.685)	(1.108–1.967)		
Accidents/injuries	2.497	2.095	1.430	2.662
	(2.047–3.046)	(1.374–3.195)	(1.189–1.720)	(2.084–3.399)
Long-standing health problem				1.6210 (1.393–1.884)
BMI category			1.140	1.114
			(1.055–1.231)	(1.015–1.222)
Binge drinking			0.887	
			(0.787–0.999)	
Social support (help from neighbours)	1.064			
	(1.015–1.115)			

RS = Serbia, BG = Bulgaria, NO = Norway, FI = Finland. NA = Not available. Table 5 presents only those factors from the post-selection logistic regression models that reached statistical significance at *p* ≤ 0.05. Complete model output for all variables entered into the second-stage logistic regression (including non-significant factors) is available in Supplementary Tables S4–S7.

## Data Availability

The data used in this study were obtained from the Eurostat microdata access programme under approval No. RPP 279/2025-EHIS (1 December 2025). Restrictions apply to the availability of these data, which were obtained from Eurostat and are available to qualified researchers upon application at https://ec.europa.eu/eurostat/web/microdata/european-health-interview-survey (accessed on 12 August 2026).

## Supplementary Materials

The following supporting information can be downloaded at https://www.mdpi.com/article/10.3390/healthcare14193242/s1, Supplementary Table S1: Missing data by countries; Supplementary Table S2: Characteristics of respondents with health status and social determinants according to pain status, all countries; Supplementary Table S3: LASSO-selected logistic regression coefficients for presence of pain, by country; Supplementary Table S4: Logistic regression model of factors associated with pain—Serbia (RS); Supplementary Table S5: Logistic regression model of factors associated with pain—Bulgaria (BG); Supplementary Table S6: Logistic regression model of factors associated with pain—Norway (NO); Supplementary Table S7: Logistic regression model of factors associated with pain—Finland (FI).

## Abbreviations

The following abbreviations are used in this manuscript:

ANOVA	Analysis of Variance
AUC	Area under the receiver operating characteristic curve
BG	Bulgaria
BMI	Body mass index
CI	Confidence interval
EHIS	European Health Interview Survey
EU	European Union
FI	Finland
IASP	International Association for the Study of Pain
LASSO	Least Absolute Shrinkage and Selection Operator
NO	Norway
OR	Odds ratio
OSS-3	Oslo Social Support Scale (3-item)
RS	Republic of Serbia
SD	Standard deviation
