# Supplementary material for: Social Determinants of Pain Prevalence: A Cross-National Comparison of Four European Countries
Source: Healthcare (Basel). 2026 Oct 1;14(19):3242. doi: 10.3390/healthcare14193242 (PMC13665331; doi:10.3390/healthcare14193242)
Supplement: Supplementary file 1 [file healthcare-14-03242-s001.zip › healthcare-4542498-supplementary.pdf]

Supplementary Table S1. Missing data by countries

Variables	RS n=13,178	BG n=7,540	NO n=7,913	FI n=6,251
Age / age group, n (%)	0 (0,0%)	0 (0,0%)	0 (0,0%)	0 (0,0%)
Gender, n (%)	0 (0,0%)	0 (0,0%)	0 (0,0%)	0 (0,0%)
Education level, n (%)	8 (0,1%)	2 (0,0%)	288 (3,6%)	0 (0,0%)
Labour status, n (%)	22 (0,2%)	4 (0,1%)	38 (0,5%)	86 (1,4%)
Marital status, n (%)	26 (0,2%)	17 (0,2%)	8 (0,1%)	64 (1,0%)
Household size, n (%)	0 (0,0%)	0 (0,0%)	0 (0,0%)	62 (1,0%)
Household income, n (%)	0 (0,0%)	277 (3,7%)	7 (0,1%)	713 (11,4%)
Degree of urbanization, n (%)	NA	0 (0,0%)	0 (0,0%)	0 (0,0%)
Self-rated health, n (%)	718 (5,4%)	309 (4,1%)	5 (0,1%)	394 (6,3%)
Long-standing health problem, n (%)	12 (0,1%)	6 (0,1%)	2 (0,0%)	101 (1,6%)
Daily life limitations, n (%)	13 (0,1%)	4 (0,1%)	17 (0,2%)	190 (3,0%)
Cardiovascular diseases, n (%)	26 (0,2%)	23 (0,3%)	1 (0,0%)	1,036 (16,6%)
Chronic respiratory diseases, n (%)	9 (0,1%)	14 (0,2%)	1 (0,0%)	1,011 (16,2%)
Musculoskeletal diseases, n (%)	12 (0,1%)	42 (0,6%)	1 (0,0%)	933 (14,9%)
Endocrine and metabolic diseases, n (%)	67 (0,5%)	148 (2,0%)	1 (0,0%)	957 (15,3%)
Mental health diseases, n (%)	11 (0,1%)	55 (0,7%)	1 (0,0%)	910 (14,6%)
Genitourinary diseases, n (%)	12 (0,1%)	57 (0,8%)	1 (0,0%)	940 (15,0%)
Accidents and injuries, n (%)	9 (0,1%)	1 (0,0%)	13 (0,2%)	269 (4,3%)
BMI category, n (%)	1,580 (12,0%)	362 (4,8%)	191 (2,4%)	500 (8,0%)
Physical activity (excl. walking), n (%)	725 (5,5%)	313 (4,2%)	27 (0,3%)	821 (13,1%)
Daily walking duration, n (%)	1,713 (13,0%)	862 (11,4%)	2,464 (31,1%)	1,034 (16,5%)
Smoking, n (%)	3,953 (30,0%)	323 (4,3%)	25 (0,3%)	143 (2,3%)
Binge drinking, n (%)	4,701 (35,7%)	331 (4,4%)	37 (0,5%)	NA
Social support, n (%)	930 (7,1%)	349 (4,6%)	196 (2,5%)	494 (7,9%)
Pain status, n (%)	717 (5,4%)	322 (4,3%)	15 (0,2%)	444 (7,1%)
Pain intensity (last 4 weeks), n (%)	717 (5,4%)	322 (4,3%)	15 (0,2%)	444 (7,1%)
Limitation in daily activities due to pain, n (%)	719 (5,5%)	327 (4,3%)	17 (0,2%)	1,880 (30,1%)

RS = Serbia, BG = Bulgaria, NO = Norway, FI = Finland.

Supplementary Table S2. Characteristics of respondents with health status and social determinants according to pain status, all countries.

Variable	With pain (unweighted) n=15421	Without pain (unweighted) n=17963	Statistical test, p
Age, mean±SD	56.3 ± 18.1	47.6 ± 18.8	<0.001 **
Age group, n (%)			
<35 years	2259 (30.4%)	5182 (69.6%)	<0.001 ***
35–64 years	7247 (45.3%)	8754 (54.7%)	
≥65 years	5915 (59.5%)	4027 (40.5%)	
Gender, n (%)			
Male	6140 (39.0%)	9587 (61.0%)	<0.001 *
Female	9281 (52.6%)	8376 (47.4%)	
Education level, n (%)			
Low	4287 (53.1%)	3786 (46.9%)	<0.001 ***
Middle	6749 (42.8%)	9014 (57.2%)	
High	4277 (46.2%)	4978 (53.8%)	
Labour status, n (%)			
Employed	6109 (40.6%)	8928 (59.4%)	<0.001 *
Unemployed	1088 (33.3%)	2181 (66.7%)	
Retired	6163 (59.2%)	4243 (40.8%)	
Other	1985 (43.7%)	2556 (56.3%)	
Marital status, n (%)			
Never married	3700 (37.8%)	6099 (62.2%)	<0.001 *
Married	8266 (46.7%)	9452 (53.4%)	
Widowed	2306 (64.1%)	1294 (35.9%)	
Divorced	1092 (50.5%)	1069 (49.5%)	
Household size, n (%)			
One	3233 (56.6%)	2477 (43.4%)	<0.001 ***
Two	5644 (53.8%)	4854 (46.2%)	
Three and more	6508 (38.0%)	10616 (62.0%)	
Income quintile, n (%)			
1st quintile	3035 (49.8%)	3065 (50.2%)	<0.001 ***
2nd quintile	3110 (48.8%)	3261 (51.2%)	
3rd quintile	3080 (46.1%)	3608 (53.9%)	
4th quintile	2993 (44.1%)	3798 (55.9%)	
5th quintile	2664 (40.8%)	3871 (59.2%)	
Degree of urbanization, n (%)			
Cities	3800 (52.3%)	3473 (47.7%)	0.002 *

Variable	With pain (unweighted) n=15421	Without pain (unweighted) n=17963	Statistical test, p
Suburbs	3838 (50.7%)	3739 (49.3%)	
Rural areas	3262 (53.7%)	2811 (46.3%)	
Self-rated health, n (%)			
Very good and good	7339 (33.1%)	14827 (66.9%)	<0.001 ***
Fair	5316 (67.4%)	2573 (32.6%)	
Bad and very bad	2740 (83.5%)	543 (16.5%)	
Long-standing health problem, n (%)			
Yes	9905 (65.1%)	5302 (34.9%)	<0.001 *
No	5449 (30.1%)	12634 (69.9%)	
Daily life limitations, n (%)			
Not limited	9028 (35.7%)	16272 (64.3%)	<0.001 ***
Limited	4460 (78.1%)	1254 (21.9%)	
Seriously limited	1810 (82.8%)	377 (17.2%)	
Cardiovascular diseases, n (%)			
Yes	6232 (61.8%)	3853 (38.2%)	<0.001 *
No	8463 (37.9%)	13869 (62.1%)	
Respiratory diseases, n (%)			
Yes	1607 (67.2%)	784 (32.8%)	<0.001 *
No	13112 (43.6%)	16957 (56.4%)	
Musculoskeletal diseases, n (%)			
Yes	7089 (77.2%)	2097 (22.8%)	<0.001 *
No	7688 (33.0%)	15634 (67.0%)	
Endocrine and metabolic diseases, n (%)			
Yes	3456 (63.9%)	1953 (36.1%)	<0.001 *
No	11187 (41.6%)	15731 (58.4%)	
Mental diseases, n (%)			
Yes	1328 (75.8%)	423 (24.2%)	<0.001 *
No	13447 (43.7%)	17319 (56.3%)	
Genitourinary diseases, n (%)			
Yes	1703 (76.1%)	535 (23.9%)	<0.001 *
No	13037 (43.1%)	17203 (56.9%)	
Accidents and injuries, n (%)			
Yes	1456 (66.3%)	740 (33.7%)	<0.001 *
No	13798 (44.6%)	17148 (55.4%)	
BMI category, n (%)			

Variable	With pain (unweighted) n=15421	Without pain (unweighted) n=17963	Statistical test, p
Normal weight (<25)	6003 (41.3%)	8548 (58.7%)	p<0.001 ***
Overweight (25–29.9)	5829 (47.5%)	6453 (52.5%)	
Obese (≥30)	2980 (56.1%)	2337 (43.9%)	
Physical activity without walking, n (%)			
Never	8598 (47.2%)	9613 (52.8%)	<0.001 ***
1–3 days	3806 (46.2%)	4440 (53.8%)	
4–5 days	1480 (43.3%)	1941 (56.7%)	
6–7 days	1213 (40.0%)	1816 (60.0%)	
Daily walking duration, n (%)			
<30 min/day	5415 (51.0%)	5212 (49.0%)	<0.001 *
≥30 min/day	7424 (41.0%)	10688 (59.0%)	
Smoking, n (%)			
Yes	3265 (43.9%)	4179 (56.1%)	<0.001 *
No	10903 (48.8%)	11449 (51.2%)	
Binge drinking, n (%)			
Yes	4834 (39.3%)	7463 (60.7%)	<0.001 *
No	5069 (45.1%)	6169 (54.9%)	
Social support, n (%)			
Very easy	2647 (40.3%)	3923 (59.7%)	<0.001 ***
Easy	5417 (42.6%)	7288 (57.4%)	
Possible	4820 (50.6%)	4700 (49.4%)	
Difficult	1571 (54.6%)	1306 (45.4%)	
Very difficult	707 (62.2%)	430 (37.8%)	

All percentages in this table are calculated within each variable category (row), i.e. With pain % + Without pain % = 100% for every category (e.g., age <35 years: 30.4% + 69.6% = 100%), reflecting the proportion of each subgroup that experienced pain versus did not. This differs from the original table, where percentages were calculated within the With pain / Without pain columns.
RS = Serbia, BG = Bulgaria, NO = Norway, FI = Finland.
* Chi-square; ** t-test; *** Mann-Whitney U.

Supplementary Table S3. LASSO - selected logistic regression coefficients for presence of pain, by country.

Predictor	RS	BG	NO	FI
Intercept	-2.845	-2.885	-2.138	-0.585
Gender (female vs male)	0.374	0.293	0.620	0.401
Age category	0.153	0.302	-0.180	-0.272
Education level	-0.109	-0.183	0.002	0.171
Labour status: Unemployed (ref. employed)	-0.032	-0.323	-0.639	-0.545
Labour status: Retired (ref. employed)	-0.127	0.033	-0.548	-0.203
Labour status: Other (ref. employed)	-0.055	-0.378	0*	-0.556
Marital status: Never married (ref. married)	-0.408	-0.182	0*	0.127
Marital status: Widowed (ref. married)	0.172	-0.086	-0.209	-0.029
Marital status: Divorced (ref. married)	-0.034	-0.018	-0.030	-0.069
Household size	-0.068	0.021	0.084	0.068
Household income	0.060	0.035	-0.068	—
Degree of urbanization	—	0.019	0*	0.060
Self-rated health	0.665	0.800	0.497	0.689
Daily life limitations	0.432	0.814	0.575	0.915
Long-standing health problem	—	—	—	0.475
Cardiovascular diseases	0.023	0.593	0.064	—
Chronic respiratory diseases	0.110	0.171	0.204	—
Musculoskeletal disorders	1.277	1.196	1.412	—
Metabolic/endocrine disorders	0.042	0.234	0.075	—
Mental health disorders	0.215	0.898	0.337	—
Genitourinary diseases	0.326	0.377	0.136	—
Accidents and injuries	0.897	0.682	0.322	0.958
BMI category	—	0*	0.117	0.103
Smoking status	—	-0.021	0.057	0.068
Binge drinking	—	0.068	-0.099	—
Social support (help from neighbours)	0.058	0.030	0.022	0.057

Values are LASSO regression coefficients (log-odds scale). — = variable not available/modeled for that country.
RS = Serbia, BG = Bulgaria, NO = Norway, FI = Finland.

Supplementary Table S4. Logistic regression model of factors associated with pain — Serbia (RS).

Predictor	OR	95% CI	p
Gender (female vs male)	1.465	1.337–1.605	<0.001
Age category	1.180	1.060–1.314	0.003
Education level	0.887	0.822–0.957	0.002
Labour status: Unemployed	0.947	0.826–1.086	0.437
Labour status: Retired	0.842	0.725–0.978	0.024
Labour status: Other	0.919	0.781–1.082	0.309
Marital status: Never married	0.665	0.573–0.771	0.003
Marital status: Widowed	1.195	1.027–1.390	0.021
Marital status: Divorced	0.945	0.774–1.154	0.581
Household size	0.928	0.858–1.003	0.061
Household income	1.065	1.028–1.103	0.001
Self-rated health	1.944	1.783–2.120	<0.001
Daily life limitations	1.549	1.413–1.698	<0.001
Cardiovascular disease	1.027	0.922–1.145	0.626
Chronic respiratory disease	1.128	0.933–1.364	0.212
Musculoskeletal disorder	3.598	3.238–3.998	<0.001
Metabolic/endocrine disorder	1.049	0.929–1.184	0.439
Mental health disorder	1.257	0.998–1.583	0.052
Genitourinary disease	1.400	1.164–1.685	<0.001
Accidents/injuries	2.497	2.047–3.046	<0.001
Social support (help from neighbours)	1.064	1.015–1.115	0.010

n=12,124; Nagelkerke R^2=0.344; AUC=0.802
OR - Odds ratio; CI - Confidence interval.

Supplementary Table S5. Logistic regression model of factors associated with pain — Bulgaria (BG).

Predictor	OR	95% CI	p
Gender (female vs male)	1.378	1.206–1.575	<0.001
Age category	1.360	1.161–1.593	<0.001
Education level	0.811	0.731–0.900	<0.001
Labour status: Unemployed	0.698	0.548–0.889	0.004
Labour status: Retired	1.043	0.851–1.279	0.683
Labour status: Other	0.654	0.501–0.853	0.002
Marital status: Never married	0.825	0.690–0.986	0.035
Marital status: Widowed	0.872	0.707–1.075	0.200
Marital status: Divorced	0.954	0.746–1.221	0.711
Household size	1.033	0.934–1.144	0.524
Household income	1.045	0.988–1.105	0.121
Degree of urbanization	1.029	0.951–1.114	0.482
Self-rated health	2.223	1.939–2.549	<0.001
Daily life limitations	2.307	1.966–2.707	<0.001
Cardiovascular disease	1.824	1.571–2.118	<0.001
Chronic respiratory disease	1.223	0.911–1.642	0.180
Musculoskeletal disorder	3.400	2.775–4.165	<0.001
Metabolic/endocrine disorder	1.298	1.027–1.641	0.029
Mental health disorder	2.631	1.644–4.211	<0.001
Genitourinary disease	1.476	1.108–1.967	0.008
Accidents/injuries	2.095	1.374–3.195	<0.001
Smoking	0.957	0.836–1.195	0.524
Binge drinking	1.092	0.952–1.252	0.210
Social support (help from neighbours)	1.038	0.974–1.107	0.248

n=6,734; Nagelkerke R^2=0.438; AUC=0.839
OR - Odds ratio; CI - Confidence interval.

Supplementary Table S6. Logistic regression model of factors associated with pain — Norway (NO).

Predictor	OR	95% CI	p
Gender (female vs male)	1.905	1.708–2.125	<0.001
Age category	0.819	0.721–0.930	0.002
Education level	1.026	0.950–1.109	0.510
Labour status: Unemployed	0.463	0.302–0.709	<0.001
Labour status: Retired	0.563	0.459–0.691	<0.001
Labour status: Other	0.977	0.822–1.160	0.787
Marital status: Never married	1.014	0.880–1.169	0.846
Marital status: Widowed	0.768	0.575–1.026	0.074
Marital status: Divorced	0.930	0.740–1.168	0.531
Household size	1.107	1.022–1.200	0.013
Household income	0.923	0.884–0.963	<0.001
Self-rated health	1.660	1.472–1.871	<0.001
Daily life limitations	1.804	1.592–2.045	<0.001
Cardiovascular disease	1.101	0.937–1.294	0.241
Chronic respiratory disease	1.266	1.051–1.525	0.013
Musculoskeletal disorder	4.265	3.752–4.848	<0.001
Metabolic/endocrine disorder	1.118	0.943–1.326	0.200
Mental health disorder	1.444	1.161–1.795	0.001
Genitourinary disease	1.209	0.899–1.627	0.209
Accidents/injuries	1.430	1.189–1.720	<0.001
BMI category	1.140	1.055–1.231	0.001
Smoking	1.096	0.949–1.265	0.212
Binge drinking	0.887	0.787–0.999	0.049
Social support (help from neighbours)	1.030	0.978–1.085	0.268

n=7,236; Nagelkerke R^2=0.274; AUC=0.760
OR - Odds ratio; CI - Confidence interval.

Supplemental Table S7. Logistic regression model of factors associated with pain — Finland (FI).

Predictor	OR	95% CI	p
Gender (female vs male)	1.513	1.322–1.731	<0.001
Age category	0.754	0.633–0.899	0.002
Education level	1.194	1.085–1.313	<0.001
Labour status: Unemployed	0.558	0.396–0.787	0.001
Labour status: Retired	0.815	0.640–1.038	0.098
Labour status: Other	0.558	0.445–0.699	<0.001
Marital status: Never married	1.147	0.961–1.369	0.130
Marital status: Widowed	0.958	0.699–1.313	0.791
Marital status: Divorced	0.920	0.698–1.213	0.556
Household size	1.075	0.957–1.206	0.222
Degree of urbanization	1.070	0.983–1.163	0.117
Self-rated health	2.014	1.722–2.356	<0.001
Daily life limitations	2.523	2.112–3.015	<0.001
Long-standing health problem	1.620	1.393–1.884	<0.001
Accidents/injuries	2.662	2.084–3.399	<0.001
BMI category	1.114	1.015–1.222	0.022
Smoking	1,084	0,894–1,315	0.411
Social support (help from neighbours)	1,063	0,994–1,137	0.075

n=5,193; Nagelkerke R^2=0.208; AUC=0.742
OR - Odds ratio; CI - Confidence interval.
